# Designing an IoT-Based System for Monitoring Noise Levels in the Computer Science Faculty and Library of the Technological University of Panama

**DOI:** 10.3390/s23229083

**Published:** 2023-11-10

**Authors:** Juan Jose Saldana-Barrios, Edwil Aguilar, Wing Ng, Roderit Orocu

**Affiliations:** Centro Regional de Chiriquí, Facultad de Ingeniería de Sistemas Computacionales, Universidad Tecnológica de Panamá, Panama City 0819-07289, Panama; edwil.aguilar@utp.ac.pa (E.A.); wing.ng@utp.ac.pa (W.N.); roderit.orocu@utp.ac.pa (R.O.)

**Keywords:** health, hypoacusis, educational institution, sound sensors, IoT, noise pollution, Panama

## Abstract

Noise pollution is a growing problem in urban areas, and it is important to study and evaluate its impact on human health and well-being. This work presents the design of a low-cost IoT model and implementation of two prototypes to collect noise level data in a specific area of the regional center of Chiriquí, at the Technological University of Panama that can be replicated to create a noise monitoring network. The prototypes were designed using Autodesk Fusion 360, and the data were stored in a MySQL database. Microsoft Excel and ArcGIS Pro were used to analyze the data, generate graphs, and display the information on maps. The results of the analysis can be used to develop strategies to reduce noise pollution and improve the quality of life in urban areas.

## 1. Introduction

According to the World Health Organization (WHO), an estimated one million years of healthy life were lost in 2011 due to traffic-related noise in the western part of Europe alone. The primary sources of noise pollution are construction sites, manufacturing plants, and vehicular, rail, and air traffic. Other sources of noise exposure can be wind turbines and recreational and leisure activities such as listening to loud music, concerts, or participating in video game competitions. Excessive noise can cause discomfort and has been shown in several studies to increase the risk of cardiovascular disease and hypertension, sleep disorders, hearing impairment, tinnitus, and cognitive impairment [1].

To talk about noise pollution, the concept of sound needs to be presented. The Real Academia Española (RAE) defines sound as the sensation produced in the organ of hearing by the vibratory movement of bodies, transmitted by an elastic medium such as air, and noise as the inarticulate sound, usually unpleasant. Noise is generally called unpleasant or unwanted sound for the listener; however, this will depend on the sensitivity of each person, and noise pollution refers to undesirable levels of noise caused by human activity that alter the standard of living in the affected area [2].

WHO defines noise as an underestimated threat that causes several health problems either in the short or long term such as sleep disturbances, cardiovascular effects, reduced work, school performance, and so on. They also consider that noise has become one of the main environmental nuisances in the WHO European region, and people are increasingly complaining about excessive noise. Therefore, they recommend that noise levels in bedrooms for good quality sleep should be less than 30 A-weighted decibels (dB(A) or dBA) and outside, less than 40 dBA at night; and to allow good teaching and learning conditions, less than 35 dBA. In the European Union, road traffic noise exceeds 55 dBA, with 40% of the European Union population exposed; 20% are exposed to levels above 65 dBA, and more than 30% are exposed to nighttime noise levels above 55 dBA [3].

In general terms, Lopez et al. define noise as “an unpleasant and annoying sound, with excessively high levels that are potentially harmful to hearing. There are several mechanisms of exposure to a noisy environment; this can be continuous, fluctuating, intermittent, or impulsive, and it will depend on the depth and speed with which hearing loss develops, although in any of these cases, it is unfortunately irreversible” [4].

Hernandez and company comment: “The ear is the organ that guarantees communication with the environment through the recognition of the acoustic translation of nature and in the sense of displacement within it, where sounds are not only perceived but also understood and discriminated… The ear has the tensor tympani and stapes muscles in its protective function. In the presence of loud noise, both muscles contract and cause rigidity of the tympanic membrane and the ossicular chain, producing resistance to the passage of sound into the middle ear. This mechanism protects the auditory organ from intense sound stimuli. However, as a physiological phenomenon, it is only for sounds typical of nature”. They also report that, with technological advances, noises reach high frequencies exceeding the harmful thresholds in most cases, which causes some degree of hearing impairment in the short or long term [5].

With health being an issue of vital importance and one of the Sustainable Development Goals (SDGs) established by the United Nations (UN), this study will focus on determining whether there is noise pollution in the Faculty of Computer Systems Engineering and the library of the regional center of Chiriquí of the Technological University of Panamá (UTP), and what the noise levels detected on certain times of the day are, through the design and construction of a prototype to measure noise using the Internet of Things (IoT) technology.

Marlon, in his work “Architecture of a noise measurement system for the prevention and control of noise pollution in the area of influence of the Cayetano Heredia III Hospital in the city of Piura “, defines a noise measurement system as one that is capable of receiving measured values from an environment and that presents stable, linear data with a high degree of accuracy to allow an assessment and evaluation of the noise level to which a person is exposed” [6].

Spain’s National Institute for Safety and Health at Work (INSST) explains the units used to measure noise in a publication [7]: “The characteristic quantities that allow us to quantify noise are sound pressure and frequency. Sound pressure is related to our perception of the volume or intensity of sound and frequency is related to our perception of pitch. The sound pressure value is usually stated in terms of sound pressure level, which unit is decibels (dB). A decibel is a unit resulting from dividing the sound pressure in pascals by the reference sound pressure, that which is perceptible to the human ear, using a logarithmic scale. The human ear does not respond equally to all frequencies of the sound spectrum. Thus, a high-pitched or high-frequency sound causes a greater sensation of intensity in the ear than another sound of the same intensity, but of lower frequencies. This fact is considered when characterizing a noise by means of weighting scales. The most used is the A-weighting scale (dBA) since it is the one that most closely resembles the response of the human ear”.

An example of a generally used noise measurement system is the sound level meter, which is a device that measures the pressure variation that occurs at a specific point when a sound wave propagates, expressed in decibels (dB) and calculated using a logarithmic form. Another instrument for measuring sound, derived from the sound level meter, is the dosimeter, which gives the sound pressure level as a function of exposure time. It is used to evaluate the risks of exposure to intense sounds expressed as percentages of maximum times allowed in the 8 h working day [6].

The noise problem is increasing in Panama. This work aims to develop the first IoT-based noise monitoring system prototype. This system will be the basis for a network of noise monitors that will be installed in schools, hospitals, and other healthcare institutions in David, Chiriquí, Panama. The network can be replicable in other cities and countries.

This article is organized as follows: Section 2 reviews the related work. Section 3 describes the problem definition. Section 4 describes the proposed design for the solution. Section 5 describes the construction and deployment of the solution. Section 6 presents the results of the evaluation of the solution. Section 7 discusses the limitations, and Section 8 concludes the article.

## 2. Related Work

In recent years, several investigations have been carried out in relation to noise pollution in urban areas of different parts of the world. In studies [8,9], measurements focused on the sound levels generated by vehicular traffic were made using sound level meters, where the first study obtained data from two days (Tuesday and Saturday) from 11:00 am to 5:00 pm in a period of 5 min, and the second study performed the evaluation in a week in three periods with a duration of one hour. The research carried out by Alfie and Salinas showed a variation in the sound levels in comparison with those obtained in a previous study carried out in 2008 [10] of some streets of the Historic Center of Mexico City, where there was an urban restructuring program that contemplated the construction of pedestrian corridors aimed at promoting less automobile use. Their results showed a slight reduction in noise levels; however, the authors comment that there is still a long way to go. On the other hand, the results of the research by Zamorano et al. carried out on the Mexican border showed that in all of the studied areas, the 65 dB recommended by the WHO were exceeded; in addition, the authors comment that in Mexico, there are no laws regulating the noise generated in the cities, so they consider that it is important for the population to be aware of the risk factor and the consequences of noise exposure, and also that some solutions could be the implementation of appropriate policies and the development of new road corridors to free traffic in certain areas.

To obtain a better vision for the planning or creation of policies and measures to mitigate noise pollution, noise maps are generally used to find areas with higher noise levels and estimate the number of people affected, as proposed in the following articles [11,12]. In both cases, measurements were taken using sound level meters (AR814 and Larson Davis’s SoundTrack LxT, respectively) and global positioning systems (GPS) to position the study points in creating noise maps.

In Panama, several investigations related to noise pollution have been carried out with different approaches, such as mathematical models for noise calculation, noise monitoring using expert systems, and studies through in situ measurements.

The first approach is present in the work performed by Quintero and De Frias [13], where they evaluated four mathematical logarithmic models (FHWA from the United States, Valdivia from Chile, NMPB-Routes-96 from France, and RLS90 from Germany) and calculated the percentage error after comparing the results obtained from the models with those obtained in other studies [14,15]. Quintero and De Frias concluded that the use of the NMPB-Routes-96 model is not recommended for noise calculation in Panama, since this model presented the highest percentage of error due to the large number of corrections made.

The second approach was contemplated by Gonzalez and company in their paper “Expert system prototype based on fuzzy logic for noise monitoring in educational spaces” [16], where they developed a prototype of an expert system based on fuzzy logic for noise monitoring and an alert mechanism like a traffic light that warns about noise intensity in educational spaces. In their prototype, they used a Raspberry Pi 3B for data classification using fuzzy logic rules, Arduino Mega to control the lights of the warning mechanism, a KY-038 sound sensor, relays, and LED lamps; in addition, they surveyed 197 students to obtain data about their knowledge of the effects of noise on health and whether they consider noise as a negative factor in the good development of a class. From the study, the authors determined that elementary and high schools have higher noise levels than middle schools and universities, exceeding the average of 40 dB that is recommended in a classroom.

Most of the literature consulted in relation to noise pollution in Panama focuses on the latter approach and considers that it is not given enough importance despite the fact that it has been demonstrated in several investigations that high noise levels are detrimental to health, as shown in the following article [12], in which the authors statistically demonstrated the correlation between noise pollution levels and cardiovascular problems, hearing problems and sleep disturbances. The studies’ [14,17,18,19] measurements with sound level meters and the analysis of the data collected show that noise exceeded the levels recommended by the WHO and by the various Panamanian regulations.

## 3. Problem Definition

In developing countries, the issue of noise pollution is not given much importance and is sometimes even considered a trivial and unimportant issue because it usually does not require immediate medical attention and is not fatal [20].

WHO, in its World Hearing Report [21], states that hearing is the sense whereby we perceive the sounds of our surroundings; through hearing, we can relate to our environment, communicate with others, express our thoughts, and gain an education. According to the Hearing is Living Foundation, “More than 1.5 billion people currently experience some degree of hearing loss, which could increase to 2.5 billion by 2050. In addition, 1.1 billion young people are at risk of permanent hearing loss from listening to loud music for extended periods of time… Untreated hearing loss is the third leading cause of years lived with disability worldwide. It affects people of all ages, as well as families and economies. An estimated one trillion U.S. dollars is lost each year due to our collective failure to adequately address hearing loss. While the financial burden is enormous, what cannot be quantified is the distress caused by the loss of communication, education, and social interaction that accompanies untreated hearing loss” [22].

Hearing impairment is known as hyperacusis and is categorized as a health problem within chronic non-communicable diseases. In recent years worldwide, its incidence has increased considerably and is linked to the noisy environment of developed countries [5].

Hearing loss caused by prolonged exposure to high noise levels or noise-induced hearing loss is defined as a decrease in the hearing ability of one or both ears, either partially or totally, permanently, and cumulatively, because of exposure to environments with high-intensity, harmful noise levels greater than 85 dB over long periods of time. In noise-induced hearing loss, the inner ear cilia are damaged by loud noise. As a result, the ability of the sensing hair cells to transmit sounds to the brain is reduced; therefore, it is a type of sensorineural hearing loss. It is a health problem that is increasing in conjunction with the advancement of civilization. It starts in an insidious way and evolves progressively over time, gradually deteriorating, affecting both ears symmetrically. Like all sensorineural hearing disorders, it is an irreversible alteration. However, it can be prevented [5,23,24].

Currently, noise pollution in urban areas is one of the problems affecting the population of large cities, especially near schools or hospitals, as it causes various inconveniences to patients, teachers, students, and/or passers-by. According to the WHO, a person can be exposed to, on average, up to 85 decibels for a period of 3 h. Once exceeded, this interval can affect not only the performance in the different activities but can also have a serious impact on the people’s health [25].

Nowadays, some countries have noise maps for planning, management, and control of noise in their metropolitan areas. Considering this initiative, we intended to conduct a study in the province of Chiriquí, specifically in the School of Computer System Engineering (FISC) and in the library of the Technological University of Panama (UTP), to determine in the first instance if there are high noise levels in the area, since there are still no studies carried out in this sector.

### 3.1. Noise Level Standards for Different Urban Environments

**Figure 1:**
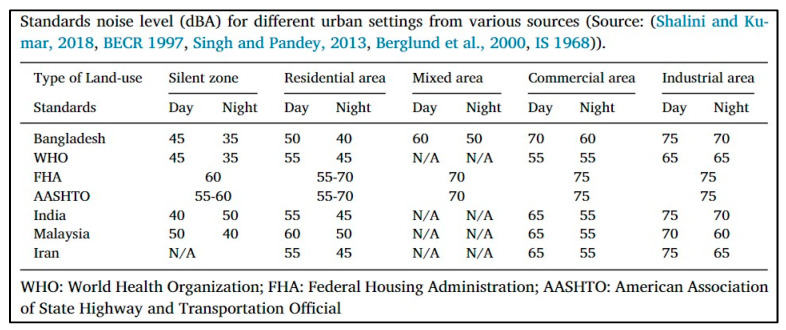
Standard noise levels (dBA) for different urban settings, from various sources [11].

Several organizations and countries have established different standards to regulate sound levels, some of which are summarized in Figure 1.

### 3.2. Allowed or Recommended Noise Ranges in Panama

A summary of the regulations governing noise in Panama is presented in Table 1.

At present, there is also a preliminary draft law 171 “which establishes measures for noise control and dictates other provisions” [27], which was presented by deputies Juan Diego Vásquez and Alina González on 25 October 2021 to the Labor, Health and Social Development Committee of the National Assembly and is at the stage of first debate for the law to be sanctioned and enacted. Article 15 establishes the sound pressure level values in the external areas of the different zones in dBA.

Noise pollution is increasing in Panama, and this work aims to develop the first IoT-based noise monitoring system network for schools, hospitals, and other healthcare institutions in the country.

## 4. Design of the Solution

This section presents an overview of the proposed solution, describing the architecture, interfaces, data model, circuit diagram, and its main hardware components dedicated to noise detection.

### 4.1. Architecture Model

**Figure 2:**
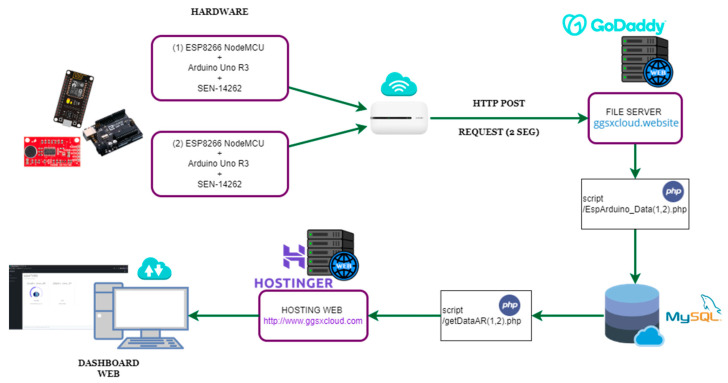
Architecture diagram. Original authorship.

Figure 2 illustrates an overview of the proposed IoT architecture, which employs two noise detection systems connected to a web server. The system has two hardware prototypes for noise pollution detection, based on an esp8266, Arduino one and a low-cost noise sensor module. A Wi-Fi network and a mobile modem are used to connect the systems with the database server and file server. The esp8266 makes HTTP post requests every 2 sec to a script hosted on the file server; this allows the data recorded by the noise sensor to be inserted into the MySQL database server, where the data are queried for real-time client-side monitoring and subsequent analysis. A detailed description of all of the hardware equipment used will be provided in Section 5.1.

### 4.2. Data Model

**Figure 3:**
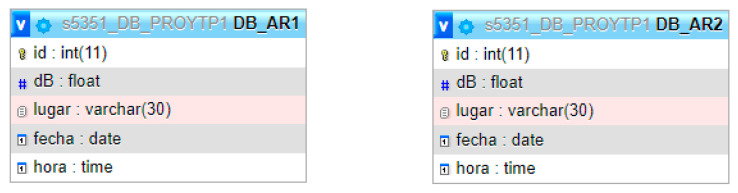
Data model. Original authorship.

The database (s5351_DB_PROYTP1) used for the project has two tables (DB_AR1 and DB_AR2) to store the measurements the two prototypes take as seen in Figure 3. The structure is the same in both tables, with five attributes: id, dB (decibels), place, date, and time. The first one is the primary key of each table; this field is auto incremented, providing a unique value to each record entered in the table. It should be noted that null or empty values are not allowed in these attributes.

### 4.3. Circuit Diagram

**Figure 4:**
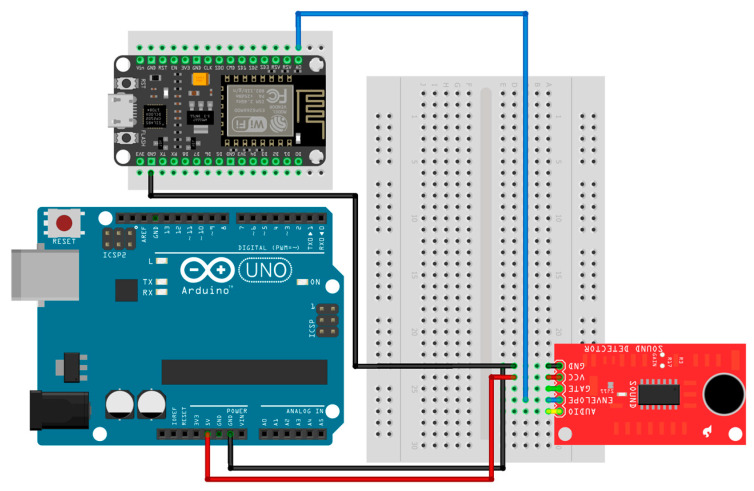
Circuit diagram. Original authorship.

The following diagram, Figure 4, shows the connections of the different internal components. The system has three main hardware components: an Arduino Uno R3, an ESP8266 board and the SparkFun SEN-14262 noise sensor. The ESP8266 is in charge of processing, collecting and sending data to the server. The SEN-14262 module is connected to the A0 pin of the ESP, powered by the Arduino Uno, which can keep the voltage stable at +5 V. Both boards have a jumper connected to the GND of the acoustic sensor to stabilize the data signal.

### 4.4. Web Interface

**Figure 5:**
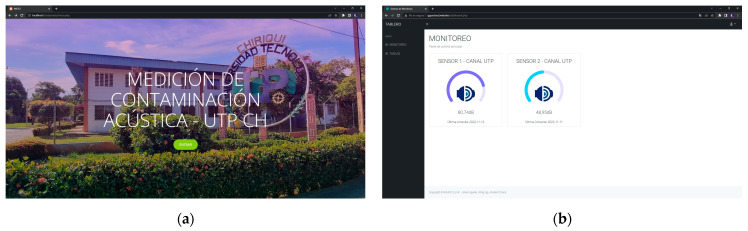
Images captured from the dashboard: (**a**) Home page to access the monitoring system; (**b**) dashboard in real-time of the data received from the sensors. Original authorship.

The development of the web interface, Figure 5, was carried out with the objective of visualizing the data captured in real-time and monitoring the operation of the noise detection system nodes. A dashboard written in PHP language (b) is used, hosted with its domain registered to be visible on the web; the data are extracted from the MySQL database and are updated at the time using AJAX on the client side. In the following illustration, one can see the web system.

## 5. Construction and Deployment

The following sections present a description of the deployment process for noise detection systems. The devices used and the deployment processes are listed.

### 5.1. Hardware Description

Table 2 provides an overview of the components and materials used in the prototype development:

### 5.2. Description of Technologies and Services Used

A 2-month contract for hosting, domains and database services was established with a cloud service provider for the implementation of the IoT architecture. These services were indispensable for the deployment of the cloud architecture proposed in Figure 2. The provider that oversaw our infrastructure was Easy Minecraft Hosting, a database provider. The file server, hosting and domains were distributed between GoDaddy and Hostinger. The fundamental service for monitoring the values recorded in our system was the web Dashboard, written in PHP language and using JS scripts to update the data in real-time.

### 5.3. Project Implementation Cost

Below are two tables that detail the costs of hardware and cloud architecture. Table 3 lists all of the hardware components used for the development of the prototype, which were purchased through the Amazon platform, so the unique ASIN code of each product is attached. The prices displayed are those obtained through Prime membership.

Table 4 lists the price of the contracted cloud services. During the development of the project, changes were made in the cloud section due to the overload of requests from the prototypes to the main hosting in GoDaddy causing crashes in the web monitoring system. Consequently, we implemented an architecture using microservice, where the monitoring system and other files were migrated to another server in Hostinger. Thanks to these changes, it was possible to stabilize the system in general, avoiding data loss and crashes in the web client. In the future, it is expected that all cloud components of the project will be migrated to Hostinger; however, it is not necessary to hire a variety of services to implement a similar architecture, as one can opt for a sufficiently robust service to avoid the acquisition of several servers. In the end, it will also depend on the scalability one wants to achieve and the economic resources available for the project.

### 5.4. Sensor Selection

Based on the integrated circuit, there were a minimum of five types of Arduino sound sensor modules:

- Based on KY-038
- Based on LM 393
- Based on LMV 324M
- Based on MAX 9814
- Based on MAX 4466.

At the beginning of the development of the project, sensor modules based on KY-038 and KY-037 were used. These incorporate a microphone together with an LM393 comparator, which allows obtaining the reading as an analog value and in digital form.

This type of non-amplified sensor usually uses the digital output to detect the sound when it exceeds a certain threshold, regulated through a potentiometer located on the board. This type of sensor based on the KY-038 is effective for sound detection, but not for measurement.

Consequently, we chose the SparkFun SEN-12642, which employs a 324M LMV comparator that not only provides an audio output but also an analog representation of its amplitude with a higher accuracy and a wider range of gain, allowing a better calibration of the amplitude. The envelope pin output allows one to easily read the amplitude of a sound by simply measuring the analog voltage, thus allowing monitoring of the ambient noise.

### 5.5. Prototype Calibration

**Figure 6:**
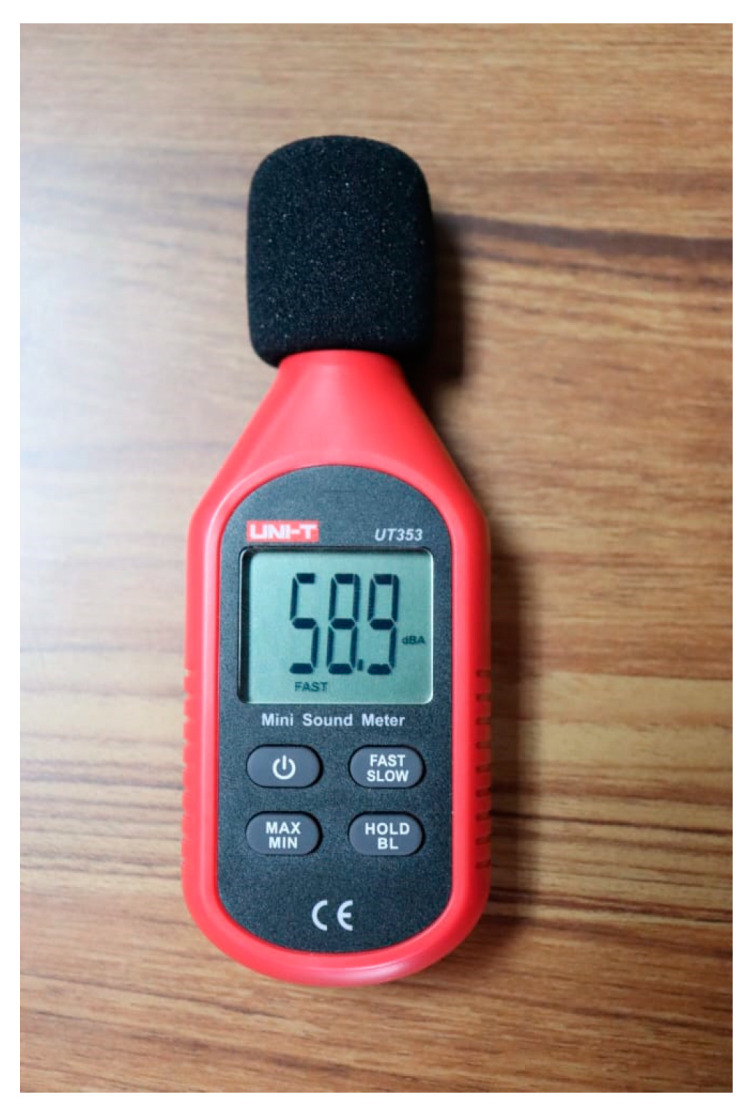
Photograph of the sound level meter used to calibrate and validate the prototype data.

For the proper functioning of the prototype, a calibration was performed using the UT353 sound level meter (Figure 6) and the DB_METER_LIBRARY library, where a sample was taken in an area with few fluctuations in sound levels and the analytical values measured by the sensor and the decibels shown by the sound level meter were compared.

DB_METER_LIBRARY is an Arduino library for implementing a dB meter with an analog sensor. One must calibrate the analog sensor with an external SPL meter and set the appropriate ANALOG_SOUND_PIN, ADC_SOUND_REF and DB_SOUND_REF values.

ANALOGO_SOUND_PIN corresponds to the pin number on which our sound sensor was connected to the ESP8266 board. ADC_SOUND_REF was the analog value of our sound module, and DB_SOUND_REF was the sound level meter value. A logarithmic equation was used to return the noise level in decibels as a result.

Result = 20 ∗ log (x/(ADC_(SOUND_REF))) + DB_SOUND_REF (1)

After 25 test measurements, the calibration showed that the sound level meter and the first prototype of the noise meter were 94% equivalent.

### 5.6. Design and Construction of the Prototype

**Figure 7:**
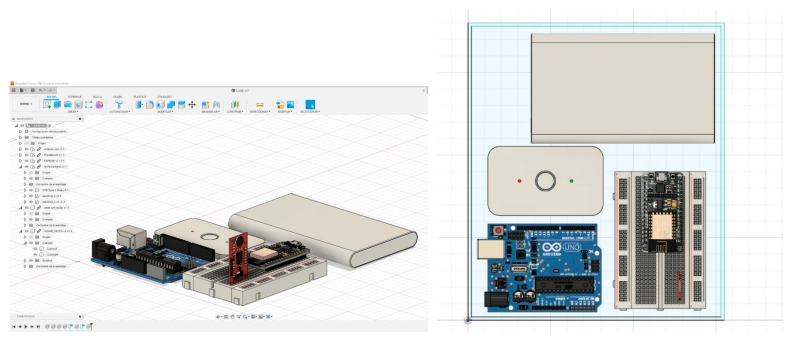
Conceptual design and 3D model of the prototype components.

Before making the physical prototype, a conceptual design (Figure 7), of the size and distribution of the components that would make up the device was made using Autodesk’s Fusion 360 tool to have a guide during manufacturing.

Real-scale 3D models of the components were used to design the size of the case where they would be located internally. After distributing the components, we continued with the design of the rest of the case and the holes for the power connections and noise detection module.

**Figure 8:**
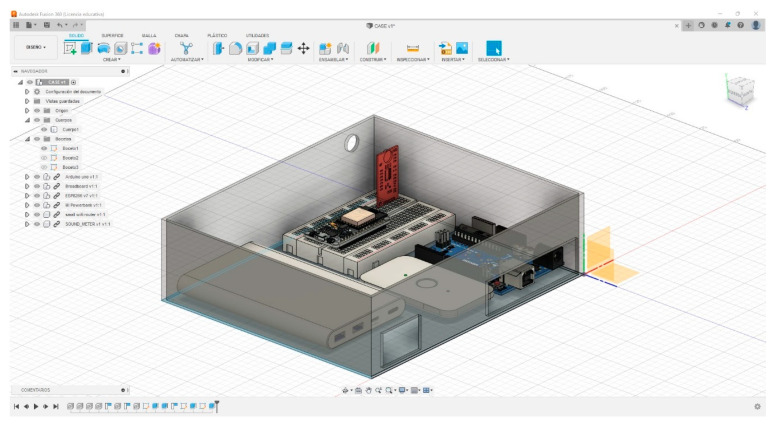
Design of the prototype housing with the components located inside it.

**Figure 9:**
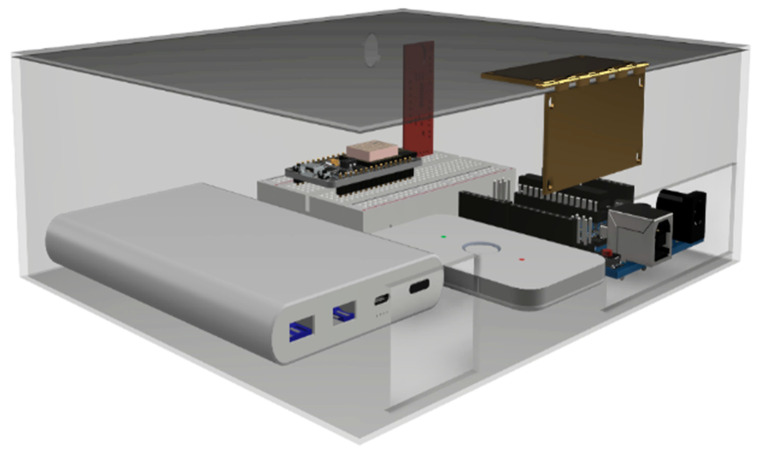
Rendering of the 3D model of the prototype to be developed.

With the 3D model of the device case, a rendering was made to visualize the possible final finish of the prototype as shown in Figure 8 and Figure 9. With the measurements taken, we proceeded to the system manufacturing.

### 5.7. Prototype Installation

**Figure 10:**
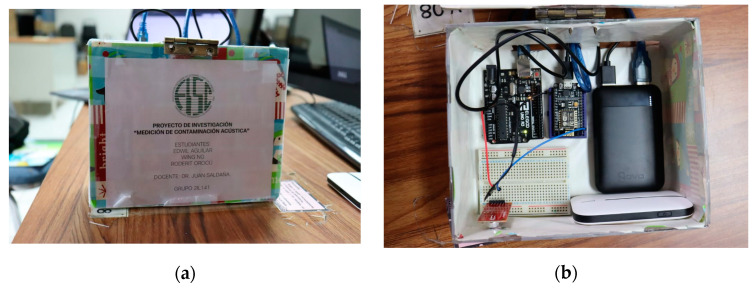
Photographs of the prototype: (**a**) case of the prototype; (**b**) photograph of the internal components of the measuring device. Original authorship.

The first step for the installation was to assemble the cases corresponding to the prototypes as shown in Figure 10. In each case were inserted the different necessary components, such as sensors, Arduino, ESP8266, and power supply, among others. After having them placed, the connections were make as shown in the circuit diagram.

The prototypes were programmed to perform the measurements with a 2 s interval, where the values to be considered for the analysis were those found in the following moments of the day:

For the first two measurement points:

- From 8:00 am to 10:00 am.
- From 12:00 pm until 2:00 pm.
- From 7:00 pm until 9:00 pm.

For the other measurement points:

- From 8:30 am to 2:30 pm.

Measurements were carried out for 5 days for the first two points and 6 days for the last two points. For the first two points, measurements were taken from 9 November to 16 November, considering only weekdays except for 10 November, since students did not have classes on that day, and from 17 November to 24 November, for the last two points, only weekdays.

### 5.8. Location

**Figure 11:**
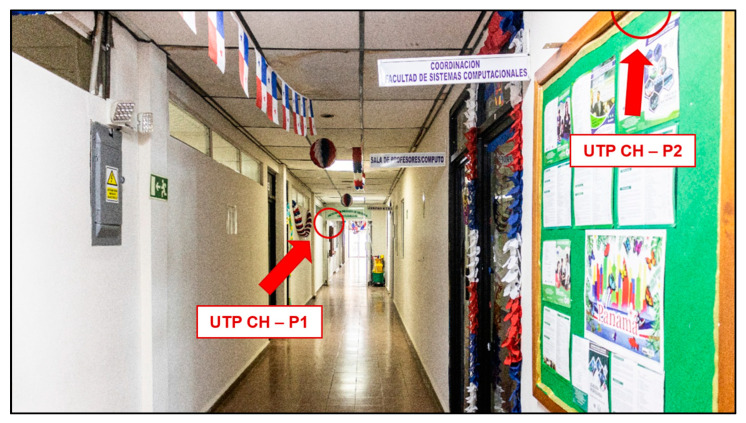
School of Computer Engineering (FISC) of the Technological University of Panama with the location of the two measurement points.

**Figure 12:**
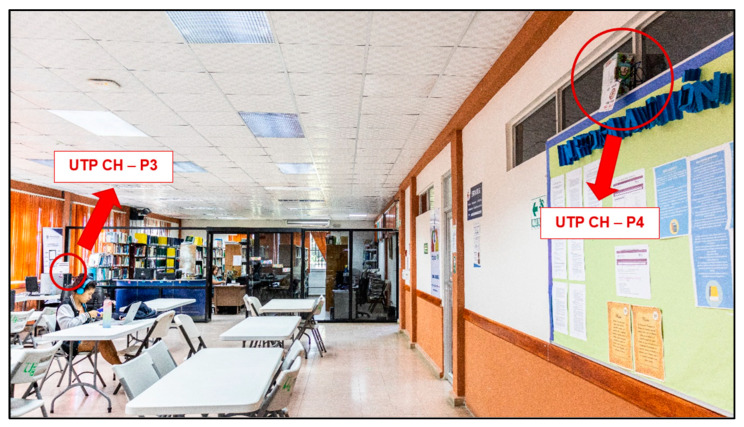
Library of the Technological University of Panama with the location of the two measurement points.

**Figure 13:**
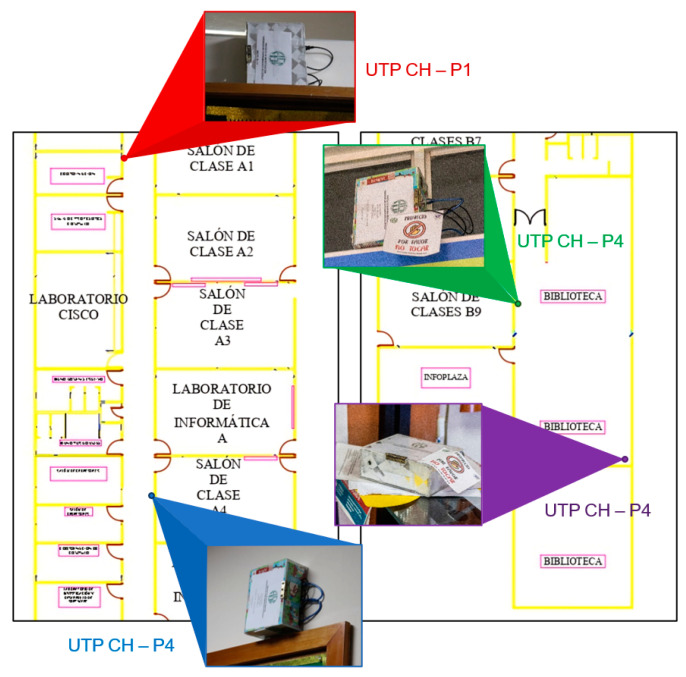
Sound level measurement points, with the location of the prototypes.

Four measurement points were taken into consideration, two of which were in the School of Computer Engineering (FISC) of the Technological University of Panamá (UTP), Chiriquí, and the other two were in the library of the UTP, Chiriquí, as shown in Figure 11, Figure 12 and Figure 13.

The left side shows the specific area selected from the FISC with the first two measurement points, and the right side shows the area of interest of the UTP library, Chiriquí, with the last two points of interest. It should be noted that only two prototypes were built, so the measurement was performed first in FISC and then in the library.

## 6. Results

Microsoft Excel was used to calculate some measures of central tendency and dispersion. In addition, graphs of interest were created with the data obtained from the measurements taken. It should be noted that for the analysis of the data, negative values were filtered, and different time intervals were selected for the different days because the negative values indicated that there was a technical failure in the prototype, and because of the failures, there were certain periods of time in which the sound levels were not measured, or the data were not saved. This is possible because the prototypes also stored and performed measurements on non-preset days.

The selected data ranges considered the following criteria:

- Cannot be a negative value
- For 9 November: 9:30 a.m. to 8:30 p.m.
- For 11 November: 8:20 a.m. to 6:35 p.m.
- For 14 November: 10:15 a.m. to 8:25 p.m.
- For 15 November: 8:15 a.m. to 10:00 p.m.
- For 16 November: 8:00 a.m. to 9:10 p.m.
- For 17 November: 10:22 a.m. to 6:42 p.m.
- For 18 November: 8:30 a.m. to 8:00 p.m.
- For 21 November: 8:51 a.m. to 8:00 p.m.
- For 22 November: 8:30 a.m. to 8:00 p.m.
- For 23 November: 9:42 a.m. to 8:00 p.m.
- For 24 November: 8:30 a.m. to 8:00 p.m.

The following Table 5, shows the measures of trend (mean, median, and mode), the measures of dispersion (standard deviation and variance), and the amount of test data used to perform the calculations.

**Figure 14:**
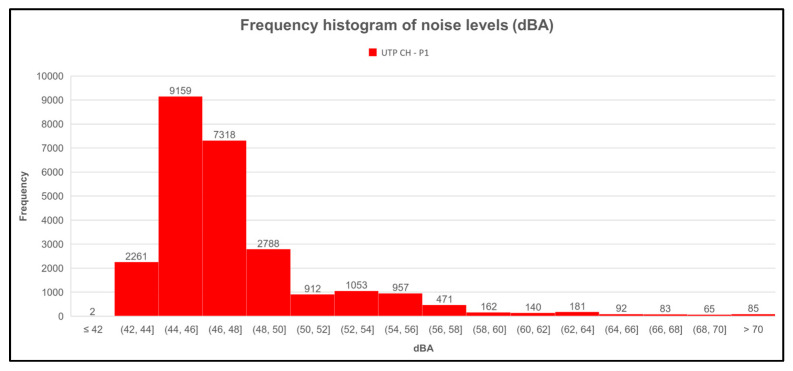
Histogram of sound level frequencies of the first measurement point (UTP CH-P1).

**Figure 15:**
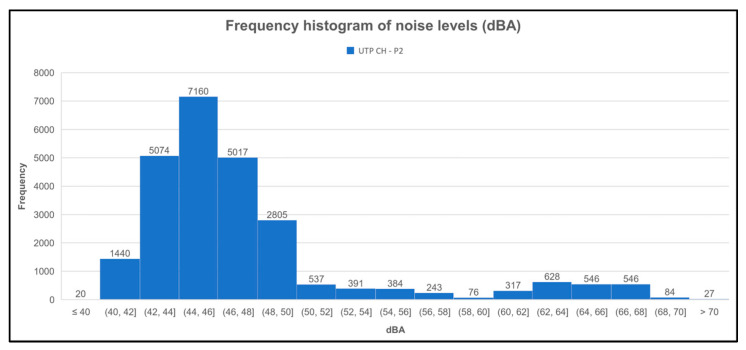
Histogram of sound level frequencies of the second measurement point (UTP CH-P2).

**Figure 16:**
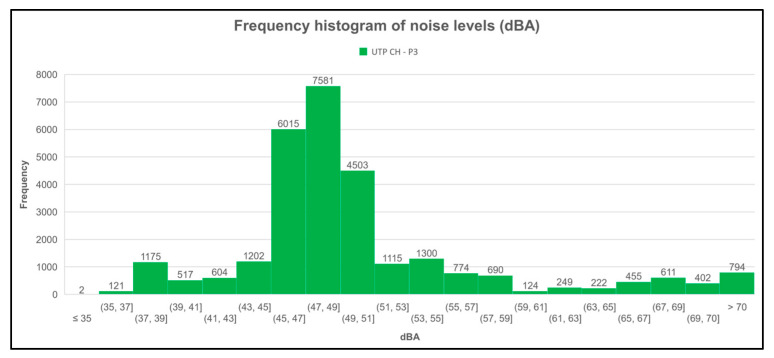
Histogram of sound level frequencies of the third measurement point (UTP CH-P3).

**Figure 17:**
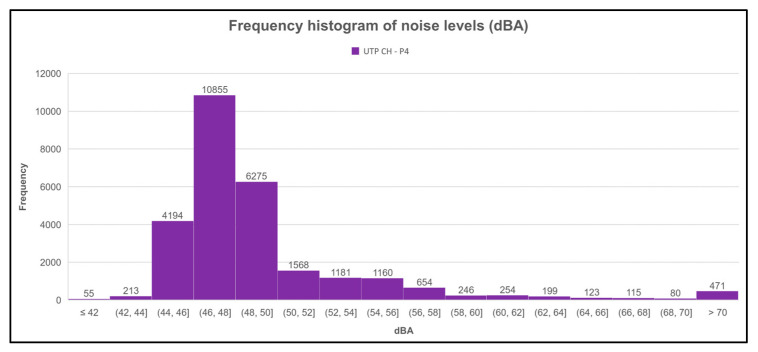
Histogram of sound level frequencies of the fourth measurement point (UTP CH-P4).

Figure 14, Figure 15, Figure 16 and Figure 17 show the frequency histograms generated with the Microsoft Excel tool for each measurement point. Frequency histograms are made to graphically summarize a set of data distributions to find a pattern or characteristics of interest. Figure 14 shows that for the first point (UTP CH-P1), which was one of those located in FISC, the range between 44 to 46 dBA ha the highest frequency, and the lowest frequency was the range of decibels below 42 dBA.

The same is observed in Figure 15 for point 2 (UTP CH-P2). The other point was also located in FISC, except that the lowest range was below 40 dBA.

For the third measurement point (UTP CH-P3), it can be observed in Figure 16 that the sound levels were usually between 45 and 51 dBA, with the range of 47 to 49 dBA the most frequent. It can also be seen that the lowest frequency of sound levels at this point was in the range of less than 35 dBA.

The behavior of the sound levels at the fourth point (UTP CH-P4) were frequently in the range of 46 to 48 dBA, with a large difference of 4580 values more than the second highest frequency interval that went from 48 to 50 dBA. The lowest frequency range for the fourth point was less than 42 dBA.

**Figure 18:**
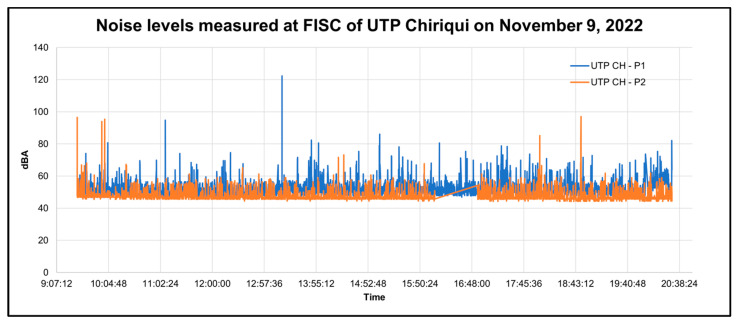
Graph of decibels measured at FISC points as a function of time on 9 November.

**Figure 19:**
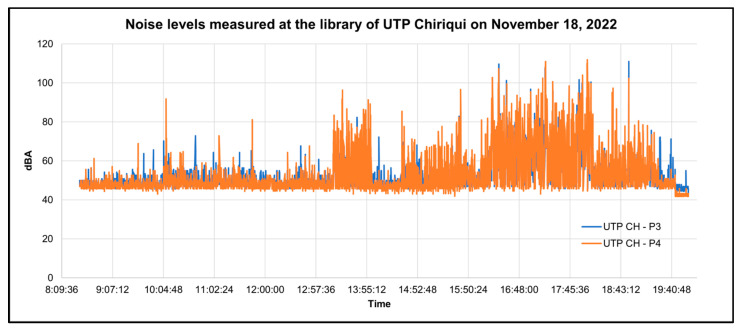
Graph of the decibels measured at points in the library as a function of time on 18 November.

In addition, for a better analysis of the behavior of the sound levels and the detection of possible technical failures as shown in Table 6, graphs were made according to the time when the decibel measurement was taken; if there was a steep slope, it indicated that no data were stored in that section. The graphs were plotted by day of measurement, and each graph shows the data from two points to facilitate the comparison of the data. Some of these graphs are shown below in Figure 18 and Figure 19.

As shown in Figure 18, the sound levels of the UTP CH-P2 usually remained constant between the values of 40 and 60 dBA, and there were few instances where the sound levels rose to the range between 80 and 100 dBA. In addition, it can be noted that the data at high sound levels did not persist over time and that there was a steep slope indicating that no data were stored in that time interval due to a technical failure of the prototype. Comparing both points, the sound levels of the UTP point CH-P1 were lower than those of the UTP point CH-P2 in most of the time periods.

In Figure 19, it can be observed that during the period between 12:57 p.m. and 7:40 p.m., high sound levels were measured. This is because, on that day, an event was held in the library due to lack of space in the university, reaching ranges between 80 dBA and 100 dBA in certain periods of time.

For better visualization, a noise map was also created using Google Earth imagery and the ArcGIS Pro tool that allowed us to create maps, include data for analysis, and display the results through maps. In the following noise map, the colors show different ranges from 47.68 dBA to 50.22 dBA and how the sound affected other sites.

**Figure 20:**
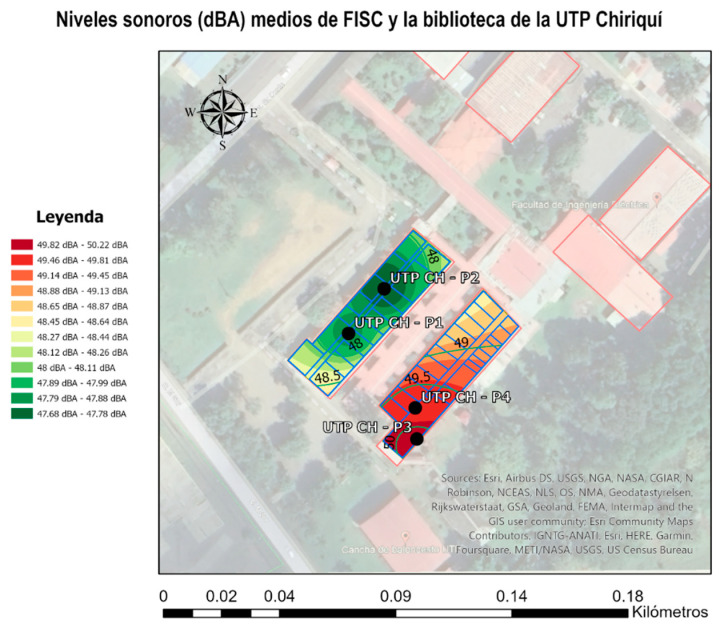
Noise map with the average sound levels of all study points obtained from the designed prototype. Original authorship.

Figure 20 shows that the UTP CH-P3 noise sensor recorded the highest reading, followed by the UTP CH-P4 sensor. This is likely because there was construction work near the library, to the south, during the measurement period. The UTP CH-P1 and UTP CH-P2 sensors were located in the next building, so the library acted as a sound barrier. Students complained about the noise near the library.

## 7. Limitations of the Solution

Like any initial product version, this prototype has technical and non-technical limitations, as described below:

### 7.1. Non-Technical Limitations

- Because the objects of study that were the focus of the project were in public places, it was necessary to request permits to place the prototypes and collect the measurements, which caused delays in the time to execute the study and the change of approaches for its realization.
- The points chosen for the evaluation of sound levels needed more stable support options for the devices; double-contact tape and small nails were used to hold the equipment to the walls.
- Devices within reach of the public may have been obstructed due to people’s curiosity and lack of supervision, which could lead to the collection of erroneous data.

### 7.2. Hardware Technical Limitations

- The prototype was equipped with a portable router for network connectivity and to send the information of the samples collected to the database server. The router had an LTE mobile network connection. This decision was made because the WIFI networks provided by the UTP had limited access to specific domains and ports that prevented connection to the project servers.
- The system required a stable power supply of +5 V throughout the circuit so that the values collected would be correct.
- At the beginning of the project, sensors KY-037 and KY-038 were used and were intended to measure changes in the sound level but did not allow continuous monitoring of sound waves in the environment. Therefore, the decision was made to replace them with the SEN-14262 sound sensor from Spark Fun.
- One of the prototype’s main challenges was portability, making a device without a computer and a direct power supply and storing measurements in the database in real-time.
- A sound level meter is usually used to measure sound levels; this is an instrument that provides objective and reproducible measurements of sound pressure levels in a standardized way. Therefore, using a particular sensor to measure sound levels was necessary.

### 7.3. Technical Limitations of Cloud Implementation

- Due to the time between measurements and sending data to the server, HTTP 502 errors were generated due to the overload of requests that our host was able to process. As a solution, we decreased the measurement time and sent the data. This affected the loss of data in real-time.
- It was decided to acquire a second web hosting server for the dashboard display to release the load of requests received by the central server that affected the data queries.
- Implementing an IoT architecture involving many cloud services, including databases, domains, and hosting, involves a high cost. Each server belongs to different providers, so prices vary.
- During the days of measurements, our database provider, for some emergency, downgraded some nodes where the systems were hosted. This caused a loss of data and forced a system out of service for some hours.

## 8. Conclusions

Noise pollution affects everyone, causing discomfort that is not initially perceived as a detrimental factor in health because people consider it to be temporary and harmless. However, they are unaware that exposure to high noise levels can cause hearing loss, which is partial or total hearing loss, a hearing impairment that affects the long term and is irremediable. However, it can be prevented by evaluating noise sources, implementing noise mitigation policies, and urban planning.

Based on these premises, it was decided to carry out a study regarding the growing severity of this problem, in this case in the city of David, province of Chiriqui, since there is scarce information regarding the degree of noise pollution in the urban region of this area. The study was conducted at the Technological University of Panama, specifically in the School of Computer Engineering and the university library, because these sections were close to a construction area. Since the location is an educational center, it is expected that the students will have an optimal environment for their classes and be able to concentrate without any source of disturbance.

The first prototype used the sensors KY-037 and KY-038, but they could not provide continuous monitoring, so building this kind of solution was not suitable. When making the prototype, it is important to ensure all components are working correctly and compare the measurements with a standardized measuring instrument so that the results are close to the expected values and follow the project objectives.

To implement the IoT system, all of the services and functions that will be used must be considered, since this will depend on the computational resources in the cloud that will be contracted. When the system exceeds the workload it can process, it is advisable to apply a replication system focused on microservices, allowing load balancing in the rest of the system, making it more stable, and avoiding measurement interruptions. On the other hand, implementing a microservices architecture will involve a higher economic cost.

With the results obtained from the data analysis, the average sound levels of the points measured in the School of Computer Engineering (FISC) and the library of the Technological University of Panama (UTP) were between 40 and 55 dBA, which is not among the levels considered harmful to health (greater than 85 dBA) and complies with the regulations established in the country: not to exceed 55 to 60 dBA. However, the average noise levels were outside the range recommended by the WHO, of less than 35 dBA, for optimal conditions for teaching and learning. Therefore, raising awareness among the university community is advised to jointly maintain the sound levels at the recommended levels so that knowledge acquisition is continuous without interruptions.

This research will be replicated to create a multi-point noise-measuring network around the university, and it can be replicated in other private and public institutions.

## Figures and Tables

**Table 1:** Comparisons of Panamanian regulations in force in laws, decrees, and resolutions [19,26].

Law No.	Decree and Resolution No.	Source	Noise in Hours (dBA)	Remarks
Law No. 66 of 10 November 1947		National Assembly		Article 88: “To dictate measures aimed at avoiding or suppressing public nuisances, such as noise, unpleasant odors, fumes, toxic gases, etc.”.
Law No. 41 of 1 July 1998		National Assembly		To regulate, through the Executive Branch, the Auditorías Ambientales and the Programas de Adecuación y Manejo Ambiental (PAMA’s).
	No. 306 of 4 September 2002	Ministry of Health	Daytime 55 dBA Evening 50 dBA	Article 4: “… within workplaces, in an eight-hour workday shall be: With constant and intense mental activity, 50 dBA maximum; Office and similar activities, 60 dBA maximum and Other work, 85 dBA”. Article 7: Industries and businesses neighboring residences or dwellings: Daytime (6:00 a.m. to 9:59 p.m.) Evening (10:00 p.m. to 5:59 a.m.) Article 11: In exclusively residential areas, it is prohibited to exceed 45 dBA (10:00 p.m. to 5:59 a.m.) and 50 dBA (6 a.m. to 9:59 p.m.). Article 13–14: Public spaces that do not harm residences, from 55 to 65 dBA maximum daytime permissible outside the premises and 55 dBA for nighttime hours. Exceeding 64 dBA is prohibited.
	No. 1 of 15 January 2004	Ministry of Health	Daytime 60 dBA Evening 50 dBA	Residential and industrial areas: Daytime (6:00 a.m. to 9:59 p.m.) Evening (10:00 p.m. to 5:59 a.m.)
	No. 506 of 6 October 1999	Ministry of Commerce and Industry	85 dBA in an 8-h workday	Provides the permissible levels of exposure in a workday of a maximum of eight (8) hours per day.
	No. 4113 of 26 June 2006	Panama Municipality	60 dBA	It is based on Executive Decree No. 1 of 15 January 2004, issued by the Ministry of Health, which determined the maximum noise level for residential and industrial areas, as well as the hourly rate.
	No. 640 of 27 December 2006	Ministry of Government and Justice		Article 12. Prohibitions in relation to vehicles in general, in numeral: f, the emission of excessive gases, noises or sounds. Article 132. It is forbidden for drivers of vehicles: numeral: s, to drive with excessive volume in the sound equipment.

**Table 2:** List of hardware components used in the prototyping process. Original authorship.

Quantity	Materials	Description
2	Arduino Uno R3	Microcontroller board based on the microchip ATmega328. It consists of a set of digital and analog I/O pins for expansion and circuitry.
2	Sensor SparkFun—SEN 14262	Audio detection board, with 3 different outputs, provides a binary indication of the presence of sound and an analog representation of its amplitude.
2	NodeMCU ESP8266	NodeMCU ESP8266 opensource development board oriented to IoT. It has 1 analog and 13 digital inputs. It is based on the ESP8266EX chip, designed to meet the needs of a connected world.
1	Router móvil Huawei	Huawei Wi-Fi Mobile is a device that allows sharing the mobile internet connection with several devices, receiving 3G/4G signals and sharing it through a hotspot.
2	Jumper Pack	The jumpers allow closing electrical circuits, of which two connections are part. It is used in test board “protoboars”, making possible the connection of two elements in this board.
2	Protoboard	It is a tool used to connect electrical components and cables together.
2	PowerBank	PowerBank is a portable external battery that can be used to charge different electronic devices through a cable with USB connection.
1	UT353 Sound Level Meter	UT353 is a mini sound meter that can convert ambient sound into electrical signals, process data and display the results on the LCD screen.

**Table 3:** Table of prices of hardware components used in making the prototypes. Original authorship.

Description	Quantity	Unit Price	Cost Total	Reference
Arduino Uno R3 Elegoo	2	USD 17.99	USD 35.98	Amazon ASIN code: B01EWOE0UU
HiLetgo ESP8266 NodeMCU	2	USD 12.69 (Pack 2 units)	USD 12.69	Amazon ASIN code: B010N1SPRK
Protoboard	2	USD 6.29 (Pack 2 units)	USD 6.29	Amazon ASIN code: B09NS36955
Sparkfun SEN-14262—Sound detector	2	USD 11.95	USD 23.90	Amazon ASIN code: B01B26UBYA
UNI-T UT353 Sound Level Meter	1	USD 17.58	USD 17.58	Amazon ASIN code: B08PP5TMS7
Dupont cable pack	1	USD 6.98	USD 6.98	Amazon ASIN code: B01EV70C78
Generic 5000 mAh PowerBank	2	USD 13.99	USD 27.98	Amazon ASIN code: B08T8TDS8S
Huawei AP WiFi	1	USD 63.00	USD 63.00	Amazon ASIN code: B07Z5LWMNZ
**TOTAL INVESTMENT**			**USD 194.4**	

**Table 4:** Price table of the contracted cloud services. Original authorship.

Description	Quantity	Unit Price	Supplier	Reference
Standard Database Server—Canada	1	USD 1.50/month	Easy Minecraft Hosting	https://easyminecrafthosting.com accessed on 30 October 2022
Linux Economic Hosting with cPanel	1	USD 10.99/month	GoDaddy	https://www.godaddy.com accessed on 30 October 2022
Premium Hosting	1	USD 23.00/year	Hostinger	https://www.hostinger.com/web-hosting accessed on 30 October 2022
Web domain	2	USD 3.99/year	GoDaddy	https://www.godaddy.com accessed on 30 October 2022
**INVESTMENT** **TOTAL**		**USD 61.96**		

**Table 5:** Trend and dispersion measures of the data obtained.

Point	Data Quantity	Mean (dBA)	Median (dBA)	Mode (dBA)	Standard Deviation (dBA)	Variance (dBA^2^)
1	25,729	47.82	46.84	45.70	4.40	19.39
2	25,295	47.67	45.7	43.2	6.17	38.03
3	28,456	50.22	47.92	47.92	7.45	55.54
4	27,643	49.59	47.92	46.84	5.67	32.14

**Table 6:** Measures of interest calculated for the sound levels at all measurement points according to date.

Date	Point	Data	Max. (dBA)	Min. (dBA)	Mean (dBA)	Median (dBA)	Mode (dBA)	Standard Deviation (dBA)	Variance (dBA^2^)
9-Nov	1	4866	122.37	46.84	51.72	49.93	49.93	4.47	19.99
	2	4981	97.09	44.49	47.45	46.84	45.70	3.26	10.64
11-Nov	1	4888	99.86	43.20	46.98	45.70	45.70	3.62	13.08
	2	4733	78.81	45.70	49.86	48.95	48.95	3.27	10.67
14-Nov	1	4441	89.51	41.82	45.37	44.49	43.20	3.51	12.30
	2	4897	114.27	2.90	52.32	45.70	43.20	10.71	114.66
15-Nov	1	6675	109.55	43.20	47.78	46.84	45.70	4.28	18.28
	2	4986	74.01	40.33	44.26	43.20	43.20	2.42	5.88
16-Nov	1	4859	78.12	43.20	47.06	45.70	45.70	3.34	11.13
	2	5698	85.12	41.82	45.02	44.49	43.20	2.67	7.13
17-Nov	3	3689	86.69	44.49	51.52	49.93	47.92	4.86	23.67
	4	3604	91.03	43.20	51.33	49.93	46.84	5.72	32.68
18-Nov	3	5072	111.04	43.20	52.31	48.95	46.84	8.27	68.47
	4	4982	111.88	41.82	52.72	47.92	46.84	10.44	108.92
21-Nov	3	4917	72.21	35.09	49.94	46.84	38.73	11.86	140.55
	4	4854	84.28	41.82	47.18	46.84	45.70	2.52	6.37
22-Nov	3	5053	90.91	41.82	50.28	47.92	46.84	7.18	51.57
	4	4919	70.92	41.82	48.78	47.92	46.84	2.59	6.71
23-Nov	3	4522	111.62	45.70	49.74	48.95	47.92	3.31	10.93
	4	4449	73.13	43.2	48.77	47.92	46.84	2.73	7.43
24-Nov	3	5203	73.72	32.98	47.90	47.92	46.84	4.10	16.78
	4	4835	83.76	44.49	49.04	48.95	46.84	2.71	7.32

## Data Availability

Not applicable.
